# Supplementary material for: Lrp10 suppresses IL7R limiting CD8 T cell homeostatic expansion and anti-tumor immunity
Source: EMBO Rep. 2024 Jul 2;25(8):23. doi: 10.1038/s44319-024-00191-w (PMC11315911; doi:10.1038/s44319-024-00191-w)
Supplement: Supplementary file 1 — Appendix [file 44319_2024_191_MOESM1_ESM.pdf]

Appendix

Table of Contents

Appendix Figure S1……………………………………………………………Page 2

Appendix Table S1……………………………………………………………Page 3

Appendix Table S2……………………………………………………………Page 6

Appendix Table S3……………………………………………………………Page 13

APPENDIX FIGURE S1

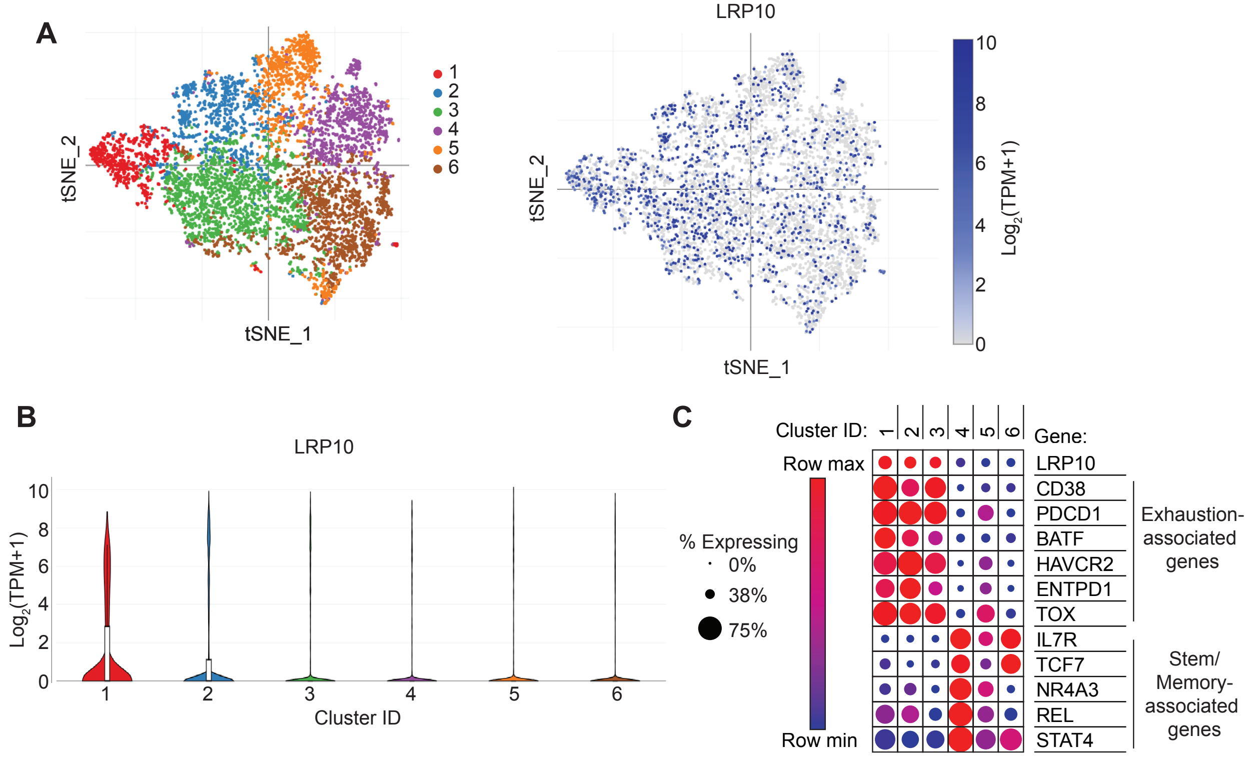

APPENDIX FIGURE S1:

A: T-distributed stochastic neighbor embedding (t-SNE) plot of CD8 TILs from melanoma patients from Sade-Feldman, et al. (Sade-Feldman *et al.*, 2018) and the distribution of Lrp10 expression. Data was visualized through the Broad Institute Single Cell Portal. Clusters 1-3 were enriched for exhaustion genes and associated with resistance to checkpoint inhibitors while clusters 4-6 were enriched for stem-memory genes and associated with checkpoint inhibitor responsiveness.

B: Violin plots showing the distribution of Lrp10 in different clusters from (A).

C: Dot plot showing overlap of Lrp10 expression with known exhaustion markers and negative correlation with stem/memory markers.

APPENDIX TABLE S1

p_val	avg_log2FC	pct.1	pct.2	p_val_adj	cluster	gene
0	1.77172289	0.432	0.045	0	0	Igfbp4
0	1.71385001	0.943	0.326	0	0	Ccr7
0	1.39997307	0.916	0.327	0	0	Sell
0	1.25708383	0.863	0.376	0	0	Fam241a
0	1.2412773	0.974	0.67	0	0	Satb1
0	1.05956569	0.917	0.552	0	0	Lef1
0	1.00338428	0.703	0.261	0	0	Cmah
0	0.95549073	0.926	0.57	0	0	Tcf7
0	0.7764086	0.996	0.951	0	0	Eef1b2
1.457E-250	0.74863569	0.962	0.805	4.705E-246	0	Rapgef6
1.349E-232	0.75815074	0.861	0.554	4.355E-228	0	Txk
3.55E-231	0.77176093	0.923	0.657	1.146E-226	0	Bach2
4.469E-231	0.80199747	0.344	0.054	1.443E-226	0	Ifngr2
9.114E-205	0.75777784	0.866	0.647	2.943E-200	0	Ppm1h
1.85E-186	0.78539873	0.581	0.268	5.972E-182	0	Actn1
0	2.56982747	0.734	0.09	0	1	Gzmk
0	2.43985142	0.975	0.711	0	1	Ccl5
8.565E-307	1.43658267	0.737	0.38	2.765E-302	1	Itga4
1.472E-302	1.37438397	0.809	0.511	4.753E-298	1	Itgb1
1.028E-290	1.90963517	0.752	0.363	3.317E-286	1	Gzmb
3.613E-286	1.01741248	0.92	0.742	1.167E-281	1	S100a10
1.539E-278	1.12866693	0.725	0.35	4.968E-274	1	Fasl
2.037E-255	1.33294429	0.593	0.196	6.577E-251	1	Pdcd1
4.026E-241	1.35180131	0.77	0.481	1.3E-236	1	Rgs1
2.14E-228	1.16175475	0.845	0.578	6.909E-224	1	Ctla2a
1.861E-142	2.53669177	0.428	0.184	6.008E-138	1	Gzma
2.05E-135	1.23058795	0.759	0.578	6.619E-131	1	Ifit3
1.528E-122	1.11684214	0.683	0.476	4.935E-118	1	Ifit1
1.139E-103	1.0408839	0.525	0.312	3.677E-99	1	Ifit3b
7.5408E-98	1.04698068	0.77	0.656	2.4345E-93	1	Isg15
0	2.08422639	0.845	0.204	0	2	Ikzf2
0	1.68254725	0.533	0.038	0	2	Klra7
1.999E-289	1.17542781	0.343	0.03	6.455E-285	2	Fcer1g
2.242E-259	0.66497488	0.29	0.021	7.238E-255	2	Klra6
2.448E-241	0.59516042	0.256	0.016	7.902E-237	2	Clnk
5.278E-227	0.91567974	0.423	0.072	1.704E-222	2	Trgv2
3.704E-207	0.92155904	0.474	0.109	1.196E-202	2	Xcl1
4.054E-117	0.63733222	0.376	0.112	1.309E-112	2	Klrc2
2.0134E-94	0.59210925	0.454	0.183	6.5003E-90	2	Pakap.1
1.7285E-92	0.68680729	0.57	0.286	5.5803E-88	2	Klhdc2
8.1598E-86	0.69521749	0.929	0.742	2.6344E-81	2	Bcl2
2.6274E-83	0.58674917	0.696	0.393	8.4824E-79	2	Nsg2
2.9875E-78	0.59375217	0.731	0.452	9.645E-74	2	Irak2
1.09E-77	0.68552947	0.6	0.328	3.5192E-73	2	Cd7
8.2232E-76	0.69586269	0.456	0.206	2.6549E-71	2	Gem
0	2.57128464	0.864	0.112	0	3	Lag3
0	2.54321909	0.749	0.057	0	3	Rgs16
0	2.25374231	0.912	0.226	0	3	Tnfrsf9

0	2.24849212	0.823	0.159	0	3	S100a4
0	2.05784889	0.84	0.179	0	3	Capg
0	1.94480791	0.479	0.039	0	3	Ifitm2
0	1.84749182	0.91	0.255	0	3	Pdcd1
0	1.67474703	0.604	0.097	0	3	Irf8
7.26E-287	1.78136441	0.872	0.303	2.344E-282	3	Cxcr6
2.16E-281	1.79089841	0.94	0.442	6.974E-277	3	Anxa2
3.041E-277	2.02544789	0.982	0.601	9.819E-273	3	Lgals1
6.488E-239	1.70644118	0.916	0.39	2.095E-234	3	Bhlhe40
4.835E-233	2.02627175	0.944	0.489	1.561E-228	3	S100a6
1.189E-224	2.69340037	0.327	0.031	3.838E-220	3	Ifitm1
4.2605E-60	3.10901042	0.376	0.142	1.3755E-55	3	Ccl4
2.051E-191	2.1958114	0.659	0.018	6.623E-187	4	Gm10501
9.9609E-34	2.64014662	0.864	0.234	3.2159E-29	4	Jun
7.9085E-31	3.34031083	1	0.518	2.5532E-26	4	Dnajb1
3.8119E-28	2.23952089	0.455	0.063	1.2307E-23	4	Id3
1.739E-17	1.93282527	0.977	0.861	5.6144E-13	4	Dnaja1
1.9572E-16	1.29949508	0.75	0.286	6.3188E-12	4	Swt1
2.0616E-16	1.49969373	0.477	0.109	6.6559E-12	4	Bag3
1.6344E-14	1.36819084	0.705	0.294	5.2766E-10	4	Ccdc117
7.5517E-14	1.3082294	0.636	0.244	2.4381E-09	4	P4ha1
1.8584E-13	1.71381344	0.864	0.53	5.9999E-09	4	Ppp1r15a
2.5477E-13	1.16973686	1	0.999	8.2254E-09	4	Ubb
1.1136E-12	1.36421566	0.773	0.427	3.5954E-08	4	Cacybp
1.4132E-12	1.69994587	0.955	0.688	4.5626E-08	4	Klf2
4.4997E-11	1.48491228	0.682	0.3	1.4527E-06	4	Fos
3.5133E-09	1.21725305	0.864	0.682	0.00011343	4	Klf6

APPENDIX TABLE S1: Table showing gene names, p-values, and average log_2 fold change for the top 15 markers for each cluster shown in Fig.5. Graphically depicted in the heatmap (Fig. 5B). Based on these markers, we defined the following clusters: 0) naïve/memory, 1) effector/exhausted, 2) innate-like, and 3) exhausted.

APPENDIX TABLE S2

Gene	p_val	avg_log2FC	pct.1	pct.2	p_val_adj
Hexb	2.683E-111	1.18035919	1	0.998	8.663E-107
Fgfr2	1.637E-83	1.19668487	0.901	0.545	5.285E-79
Lrp10	4.6956E-64	0.90923377	0.664	0.244	1.516E-59
Gm11808	3.6298E-63	0.80870946	0.929	0.799	1.1719E-58
Afm	2.5028E-59	0.81199045	0.625	0.232	8.0802E-55
Gm48099	1.5149E-58	0.89579535	0.959	0.818	4.8908E-54
Zc3h7a	3.8712E-58	0.77150681	0.983	0.904	1.2498E-53
Gm19951	2.0915E-54	0.83689699	0.987	0.915	6.7523E-50
Lars2	1.2083E-51	0.75005491	0.998	0.971	3.901E-47
Filip1l	1.0312E-44	0.64210864	0.985	0.934	3.3293E-40
Gphn	7.7208E-43	0.83718416	0.981	0.908	2.4926E-38
Jarid2	1.8171E-42	0.76503294	0.899	0.701	5.8665E-38
Cmss1	1.0768E-41	0.53353328	1	1	3.4764E-37
Camk1d	5.857E-35	0.87046154	0.989	0.943	1.8909E-30
Gucy1b2	2.8283E-34	0.46945478	0.36	0.107	9.1312E-30
Cd52	3.0589E-34	-0.66371671	0.976	0.987	9.8756E-30
Il31ra	3.0589E-33	0.72695806	0.981	0.944	9.8758E-29
Fos	1.9991E-31	0.65460845	0.362	0.115	6.4541E-27
Tmsb4x	2.142E-30	-0.54666184	1	0.999	6.9154E-26
Ddx5	5.676E-29	0.48404939	1	0.965	1.8325E-24
Ctsw	4.1443E-27	-0.57773089	0.921	0.943	1.338E-22
Tox	1.0703E-26	0.86280141	0.585	0.331	3.4553E-22
Gm21887	2.4495E-26	0.4954555	0.358	0.131	7.9082E-22
Malat1	3.8045E-25	0.41642978	0.991	0.998	1.2283E-20
Gapdh	5.7515E-25	0.60617852	0.981	0.935	1.8569E-20
Hdac9	1.3498E-24	0.40088969	0.33	0.117	4.358E-20
Srsf5	2.3282E-24	0.57524663	0.803	0.613	7.5166E-20
Myl6	2.5373E-24	-0.44478593	0.976	0.978	8.1918E-20
Trav7d-2	1.4161E-23	0.64731129	0.156	0.026	4.572E-19
Dgkb	2.2162E-23	0.43142541	0.358	0.143	7.1551E-19
AY036118	1.6402E-22	0.38687641	0.998	0.985	5.2953E-18
Capg	8.3057E-22	-0.97205063	0.281	0.504	2.6815E-17
Actb	1.3078E-21	-0.37720914	1	1	4.2222E-17
Vps37b	2.0681E-21	0.44922952	0.979	0.954	6.677E-17
Prkch	1.1758E-20	0.48664689	0.955	0.92	3.7961E-16
Dock2	1.9989E-20	0.37082909	0.991	0.973	6.4534E-16
Gm20400	7.7327E-19	0.71054319	0.42	0.211	2.4965E-14
Itga4	7.9092E-19	0.43091992	0.852	0.583	2.5535E-14
Rnaset2b	2.4888E-18	0.43883629	0.732	0.529	8.0351E-14
Pcbp2	6.3436E-18	0.39612184	0.848	0.651	2.048E-13
Sh3bgrl3	4.5894E-17	-0.45577373	0.949	0.956	1.4817E-12
Cox4i1	7.4009E-17	-0.33492243	0.957	0.957	2.3894E-12
Pfn1	2.268E-16	-0.35002174	1	0.998	7.3223E-12
Dlgap1	4.2509E-16	0.26152319	0.266	0.104	1.3724E-11
Trav12-3	7.2185E-16	0.66726476	0.148	0.038	2.3305E-11
Coro1a	9.1943E-16	-0.29945049	0.981	0.984	2.9684E-11
Dusp1	1.768E-15	0.580467	0.578	0.375	5.7079E-11
Gm48719	3.2217E-15	0.27317446	0.31	0.137	1.0401E-10

Plac8	3.9612E-15	-0.75631524	0.51	0.667	1.2789E-10
Gm47283	4.7312E-15	0.42526206	0.373	0.187	1.5275E-10
Btf3	5.1474E-15	-0.30119039	0.949	0.954	1.6619E-10
Pde1c	1.0071E-14	0.38395941	0.722	0.525	3.2515E-10
Ifitm1	1.9294E-14	-1.8565712	0.043	0.19	6.2292E-10
Socs3	2.5568E-14	0.50827748	0.505	0.313	8.2548E-10
Cox5a	3.0933E-14	-0.41548959	0.728	0.765	9.9868E-10
Klrk1	3.2625E-14	-0.54550069	0.604	0.742	1.0533E-09
Ubc	3.7413E-14	0.30331717	0.994	0.959	1.2079E-09
Jaml	4.8521E-14	-0.58873253	0.407	0.585	1.5665E-09
Cd3d	5.447E-14	-0.31277844	0.983	0.969	1.7586E-09
AW112010	7.7038E-14	-0.45208169	0.985	0.977	2.4872E-09
Lncpint	1.5079E-13	0.46352544	0.692	0.502	4.8681E-09
H2-Q6	1.5899E-13	0.31691075	0.951	0.881	5.1329E-09
CAAA0114733	2.9619E-13	-0.54317587	0.512	0.635	9.5626E-09
Il2rg	3.4695E-13	-0.32303887	0.966	0.967	1.1201E-08
Atp5g3	3.7849E-13	-0.34977934	0.848	0.866	1.222E-08
Dnajb9	6.3169E-13	0.29934017	0.358	0.184	2.0394E-08
Naca	8.2041E-13	-0.28334301	0.974	0.962	2.6487E-08
Csf1	2.0093E-12	-0.61879679	0.062	0.201	6.4871E-08
Ppp1r15a	2.3573E-12	0.39744002	0.542	0.348	7.6104E-08
Itpkb	2.6464E-12	0.40186917	0.771	0.615	8.5438E-08
Igflr1	2.9728E-12	-0.59562724	0.253	0.408	9.5977E-08
Ywhaq	3.8636E-12	0.34530906	0.522	0.34	1.2474E-07
Il1rapl1	4.4459E-12	0.31820959	0.471	0.29	1.4354E-07
Chchd2	6.2499E-12	0.32359925	0.872	0.724	2.0178E-07
Cox8a	1.0905E-11	-0.26917468	0.97	0.971	3.5206E-07
Arhgap31	1.1861E-11	0.33701979	0.45	0.268	3.8292E-07
Trbc2	1.4812E-11	0.44685299	0.831	0.721	4.782E-07
Tuba1b	2.3502E-11	0.344925	0.495	0.323	7.5877E-07
Cxcr6	2.9765E-11	-0.55095897	0.555	0.672	9.6095E-07
Tgfb1	3.1875E-11	-0.54478278	0.677	0.754	1.0291E-06
Lyst	3.3383E-11	0.49085929	0.606	0.447	1.0778E-06
Clec2d	3.7016E-11	-0.44023893	0.638	0.755	1.1951E-06
Mef2d	3.8846E-11	0.32894135	0.752	0.573	1.2541E-06
Ifi206	4.954E-11	0.33193972	0.844	0.741	1.5994E-06
Clic1	5.014E-11	-0.30033061	0.953	0.94	1.6188E-06
Camk2b	5.024E-11	-0.51198222	0.199	0.36	1.622E-06
Klrd1	5.2261E-11	-0.47203686	0.728	0.805	1.6872E-06
Actg1	7.7369E-11	-0.27752798	1	0.999	2.4978E-06
Uba52	8.3573E-11	0.31347871	0.859	0.738	2.6982E-06
Ifitm2	8.539E-11	-0.8246471	0.099	0.238	2.7568E-06
Cnn2	9.5596E-11	-0.31095897	0.884	0.898	3.0863E-06
Ly6a	1.4583E-10	-0.47460825	0.876	0.904	4.7082E-06
Gm20663	1.6124E-10	0.30738177	0.394	0.231	5.2057E-06
Ppp1ca	1.6459E-10	-0.30513275	0.818	0.866	5.3137E-06
Gvin1	1.6803E-10	0.27796137	0.428	0.256	5.4248E-06
Kpna1	1.83E-10	0.32373367	0.642	0.491	5.908E-06
Atp5c1	2.0705E-10	-0.31062259	0.747	0.802	6.6846E-06
Brd9	2.836E-10	0.2796908	0.4	0.242	9.1561E-06

Cd6	3.1847E-10	-0.32402278	0.608	0.756	1.0282E-05
Dnajb1	3.3897E-10	0.38124011	0.621	0.439	1.0944E-05
Cebpb	3.5583E-10	0.42349526	0.57	0.4	1.1488E-05
Ccr8	4.1178E-10	-0.64104265	0.056	0.17	1.3294E-05
Ms4a4b	4.4361E-10	-0.28045898	0.996	0.995	1.4322E-05
Cd47	4.7358E-10	-0.26127842	0.942	0.954	1.529E-05
Gm15283	5.3984E-10	0.33494978	0.499	0.333	1.7429E-05
Myl12a	5.9164E-10	-0.3062795	0.923	0.915	1.9101E-05
Rgs10	6.7942E-10	-0.48967556	0.244	0.379	2.1935E-05
Serf2	8.7546E-10	-0.25191942	0.942	0.957	2.8264E-05
Gm15441	1.0082E-09	0.33162184	0.45	0.289	3.2549E-05
Ifitm3	1.022E-09	-0.83349089	0.116	0.252	3.2997E-05
Lime1	1.0775E-09	0.288887	0.405	0.255	3.4788E-05
Maf	1.1358E-09	0.42172599	0.171	0.072	3.6669E-05
Slc15a2	1.5189E-09	0.26088326	0.218	0.104	4.9038E-05
Ctla2a	1.5943E-09	-0.34274172	0.837	0.886	5.1473E-05
Il2ra	2.0073E-09	-0.36414925	0.133	0.264	6.4805E-05
Stk10	2.2673E-09	0.26757662	0.833	0.73	7.32E-05
Mxi1	2.4574E-09	0.43565677	0.593	0.444	7.9336E-05
Dip2b	2.9286E-09	0.33840721	0.664	0.504	9.4549E-05
Arhgdib	3.019E-09	-0.25567154	0.964	0.964	9.7469E-05
Clk1	4.3378E-09	0.29136133	0.769	0.628	0.00014005
Ptpn6	4.4679E-09	-0.46640945	0.4	0.508	0.00014425
Tuba4a	4.7555E-09	0.2533817	0.358	0.212	0.00015353
Tnrc6a	5.5569E-09	0.25779161	0.42	0.271	0.0001794
Bcl11b	6.2677E-09	0.25066038	0.565	0.398	0.00020235
Cmip	6.7053E-09	0.312738	0.762	0.624	0.00021648
Esyt1	1.0248E-08	-0.34207106	0.741	0.773	0.00033085
Grcc10	1.104E-08	-0.34752087	0.734	0.774	0.00035644
Csnk2b	1.1216E-08	-0.29389994	0.704	0.742	0.0003621
Klre1	1.1947E-08	-0.47354493	0.216	0.347	0.00038572
Tiparp	1.2553E-08	0.28236932	0.37	0.229	0.00040526
Gm17494	1.3588E-08	0.26981973	0.345	0.211	0.0004387
Dennd4a	1.4646E-08	0.27403921	0.968	0.943	0.00047285
Rasa3	1.6893E-08	0.29095066	0.552	0.398	0.00054539
Trbv13-1	1.6982E-08	0.5340001	0.255	0.133	0.00054827
Spata5	2.3112E-08	0.2940952	0.43	0.289	0.00074618
Cotl1	2.6345E-08	-0.28301061	0.891	0.907	0.00085056
Heca	2.7247E-08	0.28998351	0.557	0.399	0.00087968
Nme2	2.7573E-08	-0.31252259	0.679	0.747	0.00089019
Vim	2.985E-08	-0.41717832	0.797	0.855	0.00096372
Dusp5	3.0183E-08	0.31025871	0.867	0.79	0.00097445
Eif3k	3.561E-08	-0.28325676	0.775	0.818	0.00114966
Klrc1	3.7977E-08	-0.41076749	0.675	0.752	0.0012261
Lilrb4a	3.9881E-08	0.39767913	0.535	0.391	0.00128755
Pkp3	4.0012E-08	-0.3412817	0.268	0.395	0.0012918
Rnaset2a	4.0431E-08	0.25410334	0.4	0.265	0.00130532
Aopep	4.1129E-08	0.27590731	0.835	0.721	0.00132784
Abtb2	4.3379E-08	0.27015975	0.302	0.178	0.0014005
Pdcd4	4.6708E-08	0.30598259	0.829	0.703	0.00150797

Tubb4b	4.6988E-08	0.2963732	0.6	0.458	0.00151701
Runx1	4.8366E-08	0.35728217	0.458	0.311	0.00156149
Runx2	5.0636E-08	-0.27565305	0.621	0.712	0.00163479
Lcp1	5.1512E-08	-0.25676754	0.976	0.965	0.00166306
Atp5k	5.8341E-08	0.26679208	0.587	0.432	0.00188353
Tnfaip3	6.3332E-08	0.25978234	0.976	0.934	0.00204467
Epb41	6.7139E-08	0.33739701	0.794	0.658	0.00216757
Gm15564	6.9768E-08	0.27712101	0.555	0.404	0.00225245
Zfp36	6.9849E-08	0.27614648	0.375	0.234	0.00225507
Anxa1	7.9264E-08	-0.27157982	0.075	0.179	0.00255905
Eno1	8.8669E-08	0.39031157	0.844	0.735	0.00286267
Slc3a2	9.9729E-08	0.34448086	0.844	0.768	0.00321975
Tuba1a	1.0235E-07	0.36636351	0.544	0.403	0.0033044
Itga1	1.3478E-07	-0.37859391	0.223	0.345	0.00435143
Trbv19	1.4221E-07	0.41272976	0.203	0.105	0.0045911
Uqcrh	1.6995E-07	-0.25341103	0.944	0.945	0.00548681
AA467197	1.75E-07	-0.29417321	0.075	0.175	0.00564972
Bach2	1.9681E-07	0.34152026	0.591	0.449	0.00635404
Tmem50a	2.0482E-07	-0.2618294	0.818	0.852	0.00661261
Anxa2	2.2693E-07	-0.46540095	0.728	0.729	0.00732648
Lgals3	2.4561E-07	-0.51930943	0.454	0.557	0.00792954
Elmsan1	2.4655E-07	0.30186736	0.719	0.59	0.00795974
Tra2b	4.9003E-07	0.27933949	0.788	0.663	0.01582046
Arl4c	5.0455E-07	0.34258774	0.636	0.488	0.01628942
Nr4a2	6.8095E-07	0.26118473	0.608	0.469	0.02198461
Ankrd17	6.9071E-07	0.25151429	0.7	0.561	0.02229953
Itgb3	7.3664E-07	0.30403886	0.366	0.245	0.02378228
S100a10	8.1283E-07	-0.27148072	0.938	0.945	0.02624223
Ptprcap	8.3988E-07	-0.26905258	0.797	0.831	0.02711561
Cish	9.2628E-07	-0.35898618	0.411	0.513	0.02990497
Tuba1c	1.0253E-06	0.25674004	0.323	0.21	0.03310225
Ndfip1	1.1825E-06	-0.31270431	0.942	0.913	0.03817791
Cblb	1.3293E-06	0.27803664	0.914	0.86	0.04291539
Psmb9	1.5103E-06	-0.29175672	0.801	0.819	0.04875945
Kansl1	1.8298E-06	0.29157682	0.702	0.565	0.05907448
Thy1	2.2711E-06	-0.2539714	0.949	0.939	0.07332131
Nr3c1	2.557E-06	0.35964659	0.758	0.667	0.08255404
Exoc4	2.5911E-06	0.25530543	0.582	0.449	0.08365279
Gna13	2.6225E-06	0.25564542	0.775	0.649	0.08466866
Hnrnpa1	2.8562E-06	-0.28618842	0.741	0.76	0.09221286
Hist1h1c	2.9208E-06	0.29470111	0.645	0.522	0.09429777
Prkca	2.9365E-06	0.30319599	0.679	0.557	0.09480452
Gm42418	3.3005E-06	0.32536278	1	1	0.10655715
Sumo2	3.5866E-06	-0.25764797	0.906	0.897	0.11579415
Flna	3.603E-06	0.29455539	0.694	0.567	0.11632296
Krtcap2	3.8178E-06	-0.25289699	0.719	0.74	0.12325622
Trbv13-2	3.8234E-06	-0.89904512	0.146	0.231	0.12343821
Ifi27l2a	3.9682E-06	0.25413344	0.85	0.745	0.12811395
Ybx3	4.0164E-06	-0.29984606	0.619	0.672	0.12966972
Smad7	4.1415E-06	0.34373969	0.764	0.658	0.13370782

Ascc3	4.6974E-06	0.25669525	0.713	0.586	0.15165482
Ywhab	7.9201E-06	-0.28414901	0.582	0.629	0.25570165
Thada	8.0269E-06	0.26520496	0.565	0.426	0.25914869
Reep5	8.3757E-06	-0.27219599	0.745	0.769	0.2704099
AU020206	1.0884E-05	-0.28182075	0.647	0.698	0.35137989
Gm36723	1.2424E-05	-0.35423948	0.167	0.26	0.40110138
Runx3	1.302E-05	0.28200289	0.749	0.644	0.42034225
Gm4070	1.33E-05	0.26936096	0.704	0.619	0.42938984
Pgls	1.678E-05	-0.25304	0.418	0.501	0.54172678
Hopx	1.8975E-05	-0.2823611	0.764	0.774	0.61259944
Ncoa2	2.1402E-05	0.27109884	0.559	0.451	0.69096011
Zfp36l2	2.4074E-05	0.30241972	0.895	0.837	0.77721359
Mapkapk3	2.4449E-05	-0.28362324	0.478	0.561	0.78932212
Pglyrp1	2.4976E-05	-0.35469393	0.42	0.488	0.80635184
Prdx1	2.6146E-05	-0.25764803	0.679	0.697	0.84412509
Klf2	2.845E-05	0.4188493	0.443	0.332	0.91851295
Zeb1	3.0854E-05	0.31062255	0.694	0.601	0.99611214
Lgals1	3.126E-05	-0.33168313	0.914	0.881	1
Ier5l	3.1324E-05	0.26745646	0.325	0.225	1
Ripor2	3.3966E-05	0.26431585	0.615	0.491	1
Havcr2	4.2333E-05	-0.42625758	0.184	0.269	1
Irf8	4.8295E-05	-0.50527121	0.171	0.252	1
Ikzf2	4.9023E-05	0.70968604	0.338	0.255	1
Mxd1	5.9487E-05	0.25295	0.777	0.682	1
Il7r	6.4072E-05	-0.35528276	0.48	0.571	1
Fosl2	8.191E-05	0.34144184	0.743	0.647	1
Bcl2a1b	0.00012594	-0.31849005	0.687	0.725	1
S100a4	0.00012796	-0.46166643	0.482	0.532	1
Ctla2b	0.00013868	-0.28816912	0.355	0.433	1
Trbv12-2	0.00014088	-0.71097861	0.077	0.143	1
Dusp2	0.00014541	0.38417137	0.874	0.811	1
Man1a	0.00018845	0.25829064	0.475	0.386	1
Xcl1	0.00022922	-0.82495348	0.045	0.1	1
Lgals9	0.00023986	-0.27754746	0.702	0.722	1
P2ry14	0.00025688	-0.27337152	0.094	0.162	1
Rgs16	0.00027361	-0.75229007	0.246	0.319	1
Inpp4b	0.00030623	-0.2811299	0.662	0.708	1
Ldlrad4	0.00034611	-0.28187325	0.338	0.404	1
Socs1	0.00049344	-0.26164464	0.567	0.619	1
Ehd1	0.0005563	-0.2808349	0.366	0.429	1
Mt1	0.00065582	-0.26745562	0.084	0.148	1
BE692007	0.00071394	-0.28294178	0.445	0.507	1
Serpinb9	0.00094178	-0.34834196	0.657	0.699	1
Nfkbiz	0.00120496	-0.4416624	0.248	0.307	1
Lmna	0.00174588	0.33001585	0.248	0.182	1
Gzma	0.00183241	-0.77560519	0.323	0.393	1
Rgs2	0.0020225	0.2578409	0.409	0.323	1
Hilpda	0.00447101	-0.3047742	0.231	0.289	1
Resf1	0.00458448	0.27006555	0.668	0.622	1
Isg20	0.00459807	-0.29587062	0.713	0.737	1

Nfkb1	0.00467825	-0.28998706	0.668	0.689	1
Gadd45b	0.00483204	-0.39853406	0.253	0.307	1
Stx11	0.00512593	-0.25457398	0.218	0.267	1
S100a6	0.00742619	-0.33078114	0.812	0.802	1
Icos	0.00882122	-0.28206832	0.762	0.784	1
Dusp4	0.01102534	-0.33264385	0.186	0.231	1
Odc1	0.0154164	0.30818233	0.741	0.699	1
Tent5a	0.01661987	-0.26393123	0.274	0.318	1
1600014C10R	0.02599192	-0.25780498	0.495	0.504	1
Lag3	0.05563175	-0.45228357	0.456	0.476	1
Rbpj	0.09753439	-0.25152973	0.505	0.488	1
Icam1	0.10371122	-0.25284018	0.332	0.349	1
Ccr7	0.11174924	-0.50536206	0.111	0.138	1
Ccl4	0.29854592	-0.85372398	0.33	0.298	1
Cxcl10	0.45492514	-0.28932972	0.216	0.194	1

APPENDIX TABLE S2: Table showing gene names, p-values, and average log_2 fold change for the differentially Expressed Genes (DEGs) between the $Lrp10^{+/+}$ and $Lrp10^{-/-}$ in the subset of expanded cells (greater than 2 clones). Shown graphically in volcano plot (Fig. 6A).

APPENDIX TABLE S3

p_val	avg_log2FC	pct.1	pct.2	p_val_adj	cluster	gene
3.4388E-86	1.28592987	0.887	0.565	1.1102E-81	0	Gm2682
1.5333E-85	1.53374198	0.986	0.789	4.9504E-81	0	Ccl5
2.6786E-80	1.12053639	0.902	0.671	8.648E-76	0	Ssh2
1.1471E-76	1.52771319	0.555	0.101	3.7034E-72	0	Klf3
1.5085E-60	1.34183127	0.82	0.513	4.8701E-56	0	Gzmk
2.7495E-48	1.01261851	0.433	0.092	8.8766E-44	0	S1pr1
1.9725E-41	0.9914875	0.455	0.139	6.3683E-37	0	Tcf7
6.6239E-39	2.0406428	0.482	0.168	2.1385E-34	0	Gzma
2.9244E-37	1.02660259	0.554	0.3	9.4416E-33	0	Thada
5.3277E-36	1.33778158	0.461	0.177	1.72E-31	0	Klf2
3.1671E-33	1.1512802	0.781	0.6	1.0225E-28	0	Ifit3
1.2053E-30	1.06552685	0.69	0.478	3.8912E-26	0	Ifit1
2.0449E-23	1.07889729	0.537	0.348	6.6021E-19	0	Ifit3b
2.1828E-20	1.11680588	0.602	0.499	7.0473E-16	0	Gbp2
1.0136E-16	1.07669447	0.456	0.31	3.2724E-12	0	Slfn5
1.668E-134	2.29625999	0.764	0.152	5.384E-130	1	Rgs16
4.028E-133	1.59393613	0.505	0.022	1.301E-128	1	Ccr8
1.11E-126	2.17671791	0.888	0.339	3.583E-122	1	Lag3
4.278E-111	1.66721818	0.627	0.105	1.381E-106	1	Irf8
2.301E-110	1.56050683	0.517	0.051	7.429E-106	1	Tnfrsf4
1.3277E-97	1.36058508	0.602	0.11	4.2864E-93	1	Il2ra
7.1854E-89	1.22211125	0.871	0.377	2.3198E-84	1	Rbpj
5.3714E-88	1.36183808	0.841	0.319	1.7341E-83	1	Capg
7.5856E-88	1.29841901	0.478	0.062	2.449E-83	1	Csf1
2.0587E-81	1.49658241	0.898	0.45	6.6465E-77	1	Tnfrsf9
8.732E-62	1.26453251	0.826	0.421	2.8191E-57	1	S100a4
1.5739E-51	2.23934741	0.378	0.077	5.0814E-47	1	Ifitm1
2.7863E-43	1.24644881	0.933	0.717	8.9955E-39	1	Sdf4
5.6926E-25	1.5212739	0.323	0.118	1.8379E-20	1	Trbv13-1
0.00797954	2.17861879	0.368	0.29	1	1	Ccl4
2.917E-136	1.91849268	0.663	0.027	9.418E-132	2	Trav7d-2
1.9308E-65	1.9091121	0.717	0.099	6.2335E-61	2	Trbv19
3.6943E-42	0.88894445	0.446	0.054	1.1927E-37	2	Gm30211
3.1718E-38	1.65324477	0.913	0.372	1.024E-33	2	Tox
5.8594E-26	1.64331945	0.685	0.253	1.8917E-21	2	Ikzf2
1.0141E-25	0.77340186	0.413	0.077	3.2741E-21	2	Igkc
1.1896E-17	0.79832163	1	0.998	3.8405E-13	2	Hexb
1.7382E-16	0.66968055	0.609	0.216	5.6118E-12	2	Tspan3
1.0703E-14	0.83543728	0.989	0.763	3.4556E-10	2	Ifi27l2a
4.1871E-14	0.80672397	0.859	0.548	1.3518E-09	2	Il10ra
7.0542E-13	0.8291718	0.315	0.087	2.2775E-08	2	Maf
3.7325E-10	0.68719429	0.924	0.685	1.205E-05	2	Pdcd1
6.0806E-10	0.67877227	0.587	0.301	1.9631E-05	2	Gbp10
1.0885E-09	0.6690882	0.761	0.431	3.5141E-05	2	Cebpb
3.1488E-08	0.70628859	0.565	0.32	0.0010166	2	Gm15441
1.126E-103	3.01361312	0.602	0.034	3.635E-99	3	Trav14-2
3.819E-64	1.80131221	0.639	0.071	1.233E-59	3	Trbv2
3.7831E-54	1.3173626	0.361	0.023	1.2214E-49	3	Pclaf

2.6049E-42	1.61532338	0.651	0.123	8.41E-38	3	Trgv2
2.6542E-37	1.80758955	0.494	0.08	8.569E-33	3	Stmn1
1.6435E-36	1.82391664	0.566	0.112	5.306E-32	3	Mki67
1.47E-32	1.36969124	1	0.885	4.7459E-28	3	Lgals1
4.2899E-31	1.41469903	0.976	0.416	1.385E-26	3	Capg
8.3633E-30	1.41317979	0.976	0.494	2.7001E-25	3	S100a4
4.0819E-27	1.30440093	1	0.831	1.3178E-22	3	Vim
2.5588E-24	1.42930566	0.639	0.178	8.2611E-20	3	Ifitm2
1.5463E-12	1.37464955	0.928	0.813	4.9923E-08	3	Hmgb2
3.1697E-10	1.35546194	0.277	0.079	1.0233E-05	3	Hist1h1b
4.5977E-10	1.4800842	0.458	0.217	1.4844E-05	3	Cenpa
5.8463E-06	1.3293133	0.795	0.645	0.18874678	3	Tubb5

APPENDIX TABLE S3: Table showing gene names, p-values, and average log_2 fold change for the top 15 markers for each cluster in the subset of cells that had greater than 2 clones. Graphically depicted in heatmap (Fig. 6F). Based on these markers, we defined the following clusters: 0) stem/memory, 1) intermediate effector/exhausted, 2) terminally exhausted, and 3) cycling.
