# Supplementary figures and images for: Lrp10 suppresses IL7R limiting CD8 T cell homeostatic expansion and anti-tumor immunity
Source: EMBO Rep. 2024 Jul 2;25(8):23. doi: 10.1038/s44319-024-00191-w (PMC11315911; doi:10.1038/s44319-024-00191-w)

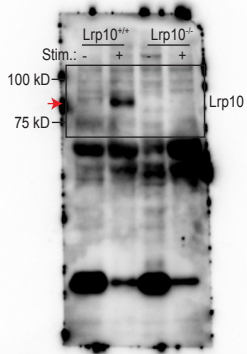

Supplement: Supplementary file 3 — Source data Fig. 1 [file 44319_2024_191_MOESM3_ESM.zip › Figure 1/Fig. 1D/Lrp10-2.pdf]

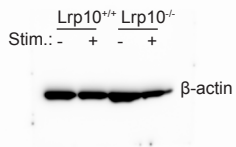

Supplement: Supplementary file 3 — Source data Fig. 1 [file 44319_2024_191_MOESM3_ESM.zip › Figure 1/Fig. 1D/b-actin.pdf]

*Lp10<sup>+/+</sup>*  
*Lp10<sup>-/-</sup>*

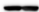

$\beta$ -actin

Supplement: Supplementary file 4 — Source data Fig. 2 Part 1 [file 44319_2024_191_MOESM4_ESM.zip › Figure 2_part 1/Fig. 2B/B actin.pdf]

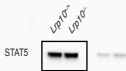

Supplement: Supplementary file 4 — Source data Fig. 2 Part 1 [file 44319_2024_191_MOESM4_ESM.zip › Figure 2_part 1/Fig. 2B/STAT5.pdf]

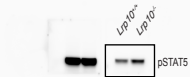

Supplement: Supplementary file 4 — Source data Fig. 2 Part 1 [file 44319_2024_191_MOESM4_ESM.zip › Figure 2_part 1/Fig. 2B/pSTAT5.pdf]

Lrp10<sup>+/+</sup>  
Lrp10<sup>-/-</sup>

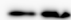

Bcl2

Supplement: Supplementary file 4 — Source data Fig. 2 Part 1 [file 44319_2024_191_MOESM4_ESM.zip › Figure 2_part 1/Fig. 2B/bcl2.pdf]

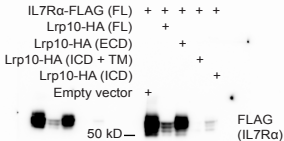

Supplement: Supplementary file 6 — Source data Fig. 3 [file 44319_2024_191_MOESM6_ESM.zip › Figure 3/Fig. 3D/FLAG-IL7R.pdf]

|                          |   |   |   |   |   |
|--------------------------|---|---|---|---|---|
| IL7R $\alpha$ -FLAG (FL) | + | + | + | + | + |
| Lrp10-HA (FL)            |   | + |   |   |   |
| Lrp10-HA (ECD)           |   |   | + |   |   |
| Lrp10-HA (ICD + TM)      |   |   |   | + |   |
| Lrp10-HA (ICD)           |   |   |   |   | + |
| Empty vector             | + |   |   |   |   |

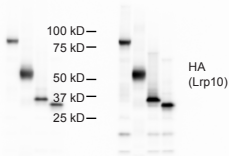

Supplement: Supplementary file 6 — Source data Fig. 3 [file 44319_2024_191_MOESM6_ESM.zip › Figure 3/Fig. 3D/Lrp1- HA.pdf]

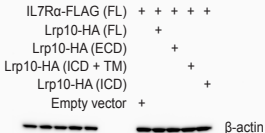

Supplement: Supplementary file 6 — Source data Fig. 3 [file 44319_2024_191_MOESM6_ESM.zip › Figure 3/Fig. 3D/B-actin.pdf]

Plasmid ratio of  
IL7R $\alpha$ -FLAG (FL) to Lrp10-HA (FL)

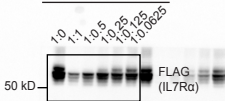

Supplement: Supplementary file 6 — Source data Fig. 3 [file 44319_2024_191_MOESM6_ESM.zip › Figure 3/Fig. 3C/FLAG IL7R.pdf]

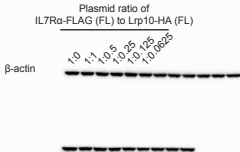

Supplement: Supplementary file 6 — Source data Fig. 3 [file 44319_2024_191_MOESM6_ESM.zip › Figure 3/Fig. 3C/b-actin.pdf]

Plasmid ratio of  
IL7R $\alpha$ -FLAG (FL) to Lrp10-HA (FL)

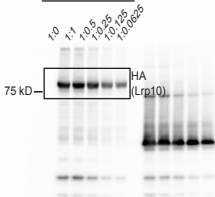

Supplement: Supplementary file 6 — Source data Fig. 3 [file 44319_2024_191_MOESM6_ESM.zip › Figure 3/Fig. 3C/HA-Lrp10.pdf]

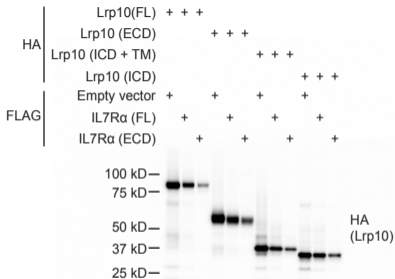

Supplement: Supplementary file 6 — Source data Fig. 3 [file 44319_2024_191_MOESM6_ESM.zip › Figure 3/Fig. 3E/Input Lrp10 HA variants.pdf]

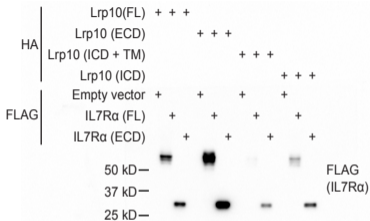

Supplement: Supplementary file 6 — Source data Fig. 3 [file 44319_2024_191_MOESM6_ESM.zip › Figure 3/Fig. 3E/INput FLAG IL7R.pdf]

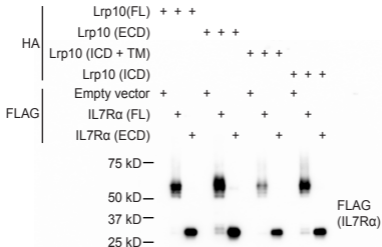

Supplement: Supplementary file 6 — Source data Fig. 3 [file 44319_2024_191_MOESM6_ESM.zip › Figure 3/Fig. 3E/IP FLAG IB FLAG.pdf]

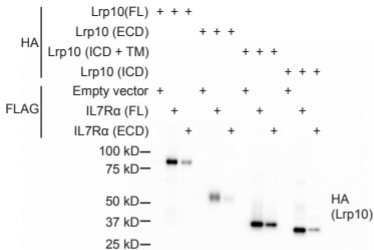

Supplement: Supplementary file 6 — Source data Fig. 3 [file 44319_2024_191_MOESM6_ESM.zip › Figure 3/Fig. 3E/IP FLAG IB HA.pdf]
