# Supplementary material for: Lrp10 suppresses IL7R limiting CD8 T cell homeostatic expansion and anti-tumor immunity
Source: EMBO Rep. 2024 Jul 2;25(8):23. doi: 10.1038/s44319-024-00191-w (PMC11315911; doi:10.1038/s44319-024-00191-w)
Supplement: Supplementary file 10 — Expanded View Figures [file 44319_2024_191_MOESM10_ESM.pdf]

Expanded View Figures

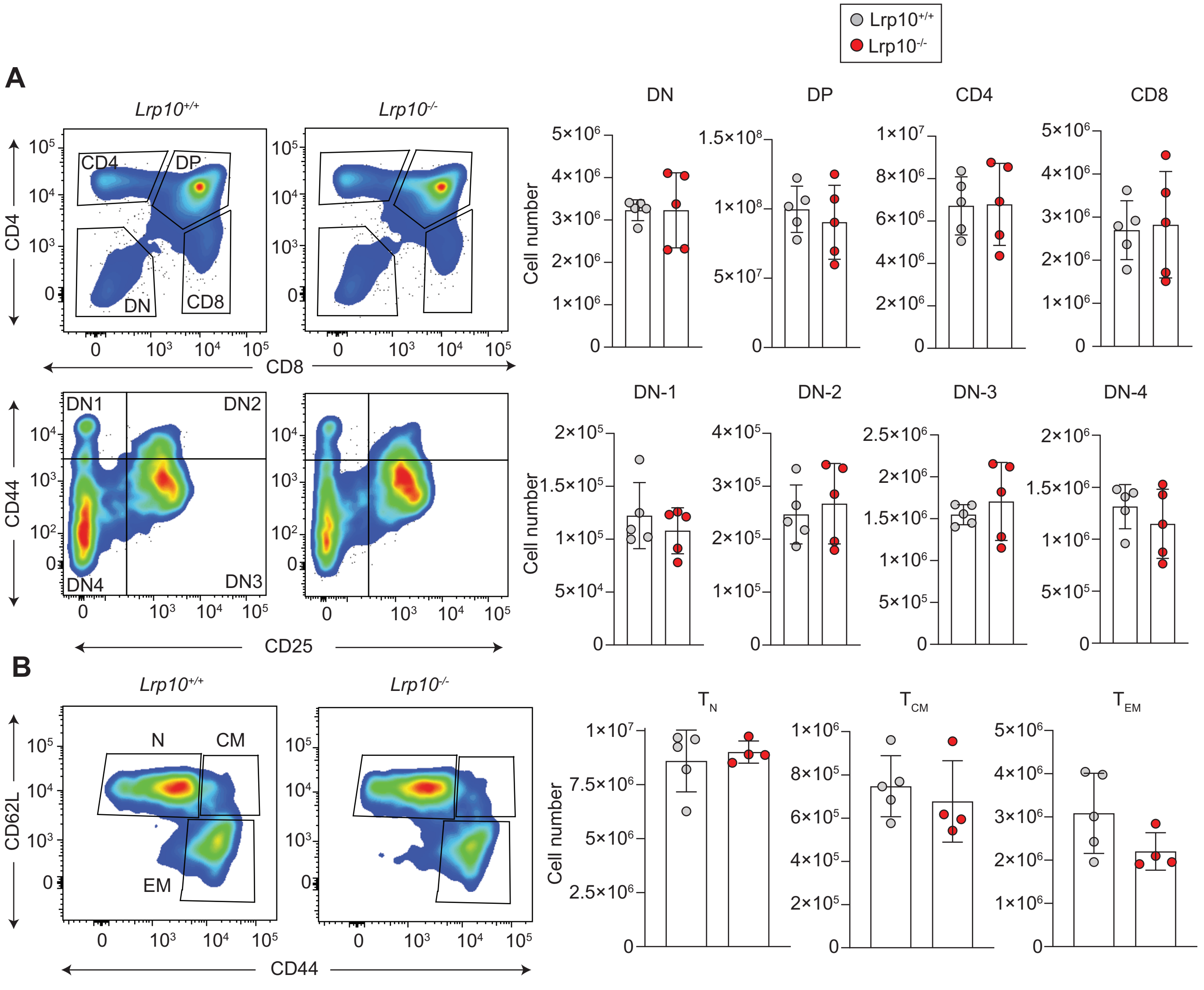

Figure EV1. Numbers of thymocyte and peripheral CD4 T cell subpopulations in *Lrp10*$^{-/-}$ mice.

(**A**) Representative FACS plots and numbers of thymic T-cell subpopulations. (**B**) Representative FACS plots and numbers of splenic CD4 subpopulations. Data information: In bar graphs, symbols represent individual mice (biological replicates), horizontal bars indicate the mean, and error bars show SD. No statistical testing was performed.

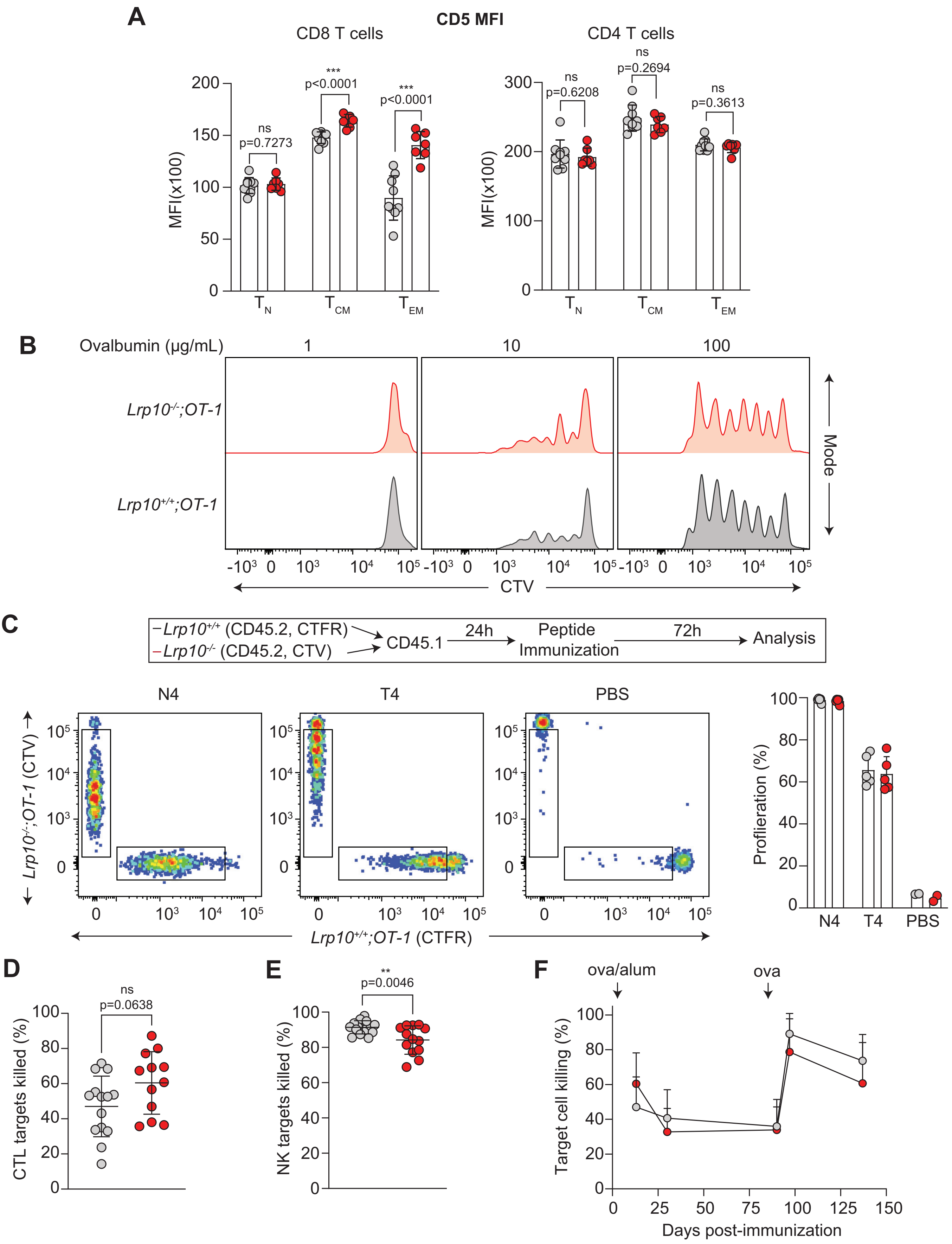
A
CD5 MFI
CD8 T cells
CD4 T cells
MFI(x100)
ns p=0.7273
*** p<0.0001
*** p<0.0001
ns p=0.6208
ns p=0.2694
ns p=0.3613
T N
T CM
T EM
B
Ovalbumin (μg/mL)
1
10
100
Lrp10-/-;OT-1
Lrp10+/+;OT-1
Mode
CTV
C
Lrp10+/+ (CD45.2, CTFR)
Lrp10-/- (CD45.2, CTV)
CD45.1
24h
Peptide Immunization
72h
Analysis
N4
T4
PBS
Lrp10-/-;OT-1 (CTV)
Lrp10+/+;OT-1 (CTFR)
Profileration (%)
D
CTL targets killed (%)
ns p=0.0638
E
NK targets killed (%)
** p=0.0046
F
ova/alum
ova
Target cell killing (%)
Days post-immunization

 © The Author(s)

Figure EV2. TCR sensitivity and cytotoxic activity in *Lrp10$^{-/-}$* mice.

(**A**) Cell surface CD5 expression in splenic CD8 and CD4 T-cell subsets. (**B**) Proliferation of *Lrp10$^{+/+}$;OT-1* or *Lrp10$^{-/-}$;OT-1* cells 72 h after co-culture with *Lrp10$^{+/+}$* dendritic cells pulsed with the indicated concentrations of ovalbumin. (**C**) 10^6 *Lrp10$^{+/+}$;OT-1* and *Lrp10$^{-/-}$;OT-1* cells were labeled with CTFR and CTV, respectively, and injected into unirradiated recipients. 24 h later mice were immunized with SIINFEKL (N4), SIITFEKL (T4), or PBS. Cell proliferation was measured 72 h after immunization based on dye dilution. (**D**) In vivo CTL cytotoxicity in *Lrp10$^{+/+}$* and *Lrp10$^{-/-}$* mice 12 days after immunization with ovalbumin and aluminum adjuvant (ova/alum). $N = 14$ *Lrp10$^{+/+}$* and $n = 12$ *Lrp10$^{-/-}$* mice. (**E**) In vivo NK cytotoxicity assay in *Lrp10$^{+/+}$* and *Lrp10$^{-/-}$* mice injected with labeled MHC-I-deficient target cells. $N = 15$ *Lrp10$^{+/+}$* and $n = 13$ *Lrp10$^{-/-}$* mice. (**F**) Serial in vivo cytotoxicity assays in the mice from (**E**) after immunization with ova/alum. Mice were given a boost of ova protein alone on day 90. $N = 14$ *Lrp10$^{+/+}$* and $n = 12$ *Lrp10$^{-/-}$* mice at each timepoint. Data information: In bar graphs, symbols represent individual mice (biological replicates), horizontal bars indicate mean values, and error bars show SD. In (**F**), symbols indicate the mean values and error bars show SD. Data from (**B**) is representative of two separate in vitro stimulation experiments. Data from (**D**, **E**) were replicated twice on separate cohorts of at least 10 mice. Data from (**C**, **F**) are from one immunization experiment each with the indicated *n*. *P* values were calculated by two-tailed unpaired *t* tests (**A**, **D**, **E**). No statistical testing was performed in (**C**, **F**). Significant *P* values were flagged as follows: $*P < 0.05$, $**P < 0.01$, $***P < 0.001$. *P* values > 0.05 were considered to be not significant (ns).

© The Author(s)

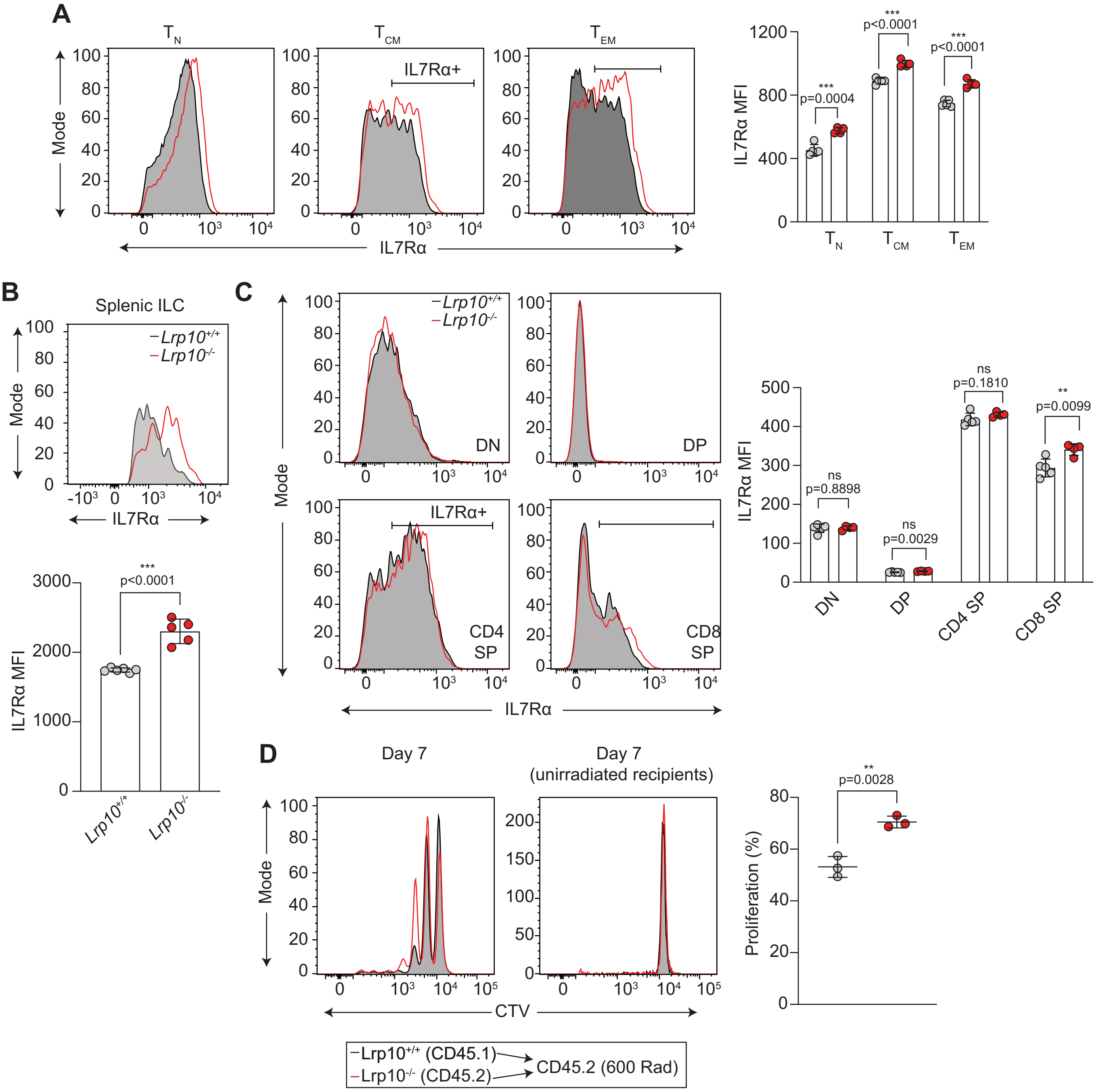

Figure EV3. IL7R expression on CD4 T cell, ILC, and thymocyte subpopulations.

(**A**) Cell surface IL7R expression in CD4 subpopulations. (**B**) Cell surface IL7R expression on Lin-CD11b-IL7R + NK1.1 + NKp46+ splenic ILCs. Lin: CD3, B220, CD11c. (**C**) Cell surface IL7R expression on thymic T-cell subpopulations. (**D**) Representative FACS plots of homeostatic expansion of *Lrp10*$^{+/+}$ and *Lrp10*$^{-/-}$ CD4 T cells labeled with CTV and injected in sub-lethally irradiated and unirradiated recipients. The bar graph shows proliferation of CD4 T cells transplanted into irradiated recipients. Proliferation was determined based on the fraction of cells that underwent at least one cell division. Data information: In bar graphs, symbols represent individual mice (biological replicates), horizontal bars indicate the mean, and error bars show SD. Data from (**D**) are from one CD4 adoptive transfer experiment with three irradiated recipients and one unirradiated recipient. *P* values were calculated by two-tailed unpaired t tests. Significant p values were flagged as follows: **P* < 0.05, ***P* < 0.01, ****P* < 0.001. *P* values > 0.05 were considered to be not significant (ns).

 © The Author(s)

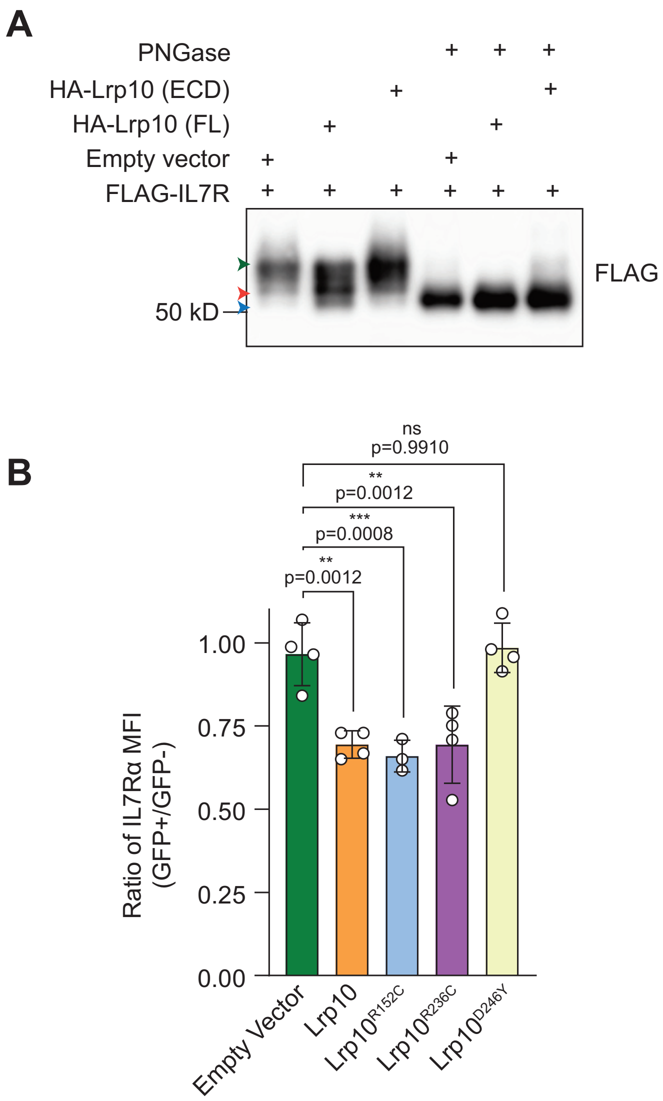

Figure EV4. Effect of Lrp10 variants on IL7R glycosylation and cell surface expression.

(**A**) IP of IL7Rα-FLAG from transfected HEK 293 T cells under denaturing conditions in the context of empty vector, Lrp10-HA (FL), or Lrp10-HA (ECD) followed by de-glycosylation with PNGase-F. (**B**) Normalized cell surface IL7R expression on activated *Lrp10*$^{-/-}$ CD8 T cells infected with MSCV retroviruses encoding GFP-only, Lrp10^{WT}-IRES-GFP, Lrp10^{R132C}-IRES-GFP, Lrp10^{R235C}-IRES-GFP, or Lrp10^{D246Y}-IRES-GFP (*chowmein* allele). IL7R levels on GFP+ cells in each sample were normalized to levels on the GFP- population. Data information: In (**B**), the symbols show the results of three or four separate retroviral transductions (biological replicates), the horizontal bars indicate mean values, and error bars show SD. *P* values were calculated by one-way ANOVA with Dunnett's multiple comparisons test. Significant *P* values were flagged as follows: *$P < 0.05$, **$P < 0.01$, ***$P < 0.001$. *P* values > 0.05 were considered to be not significant (ns).

© The Author(s)

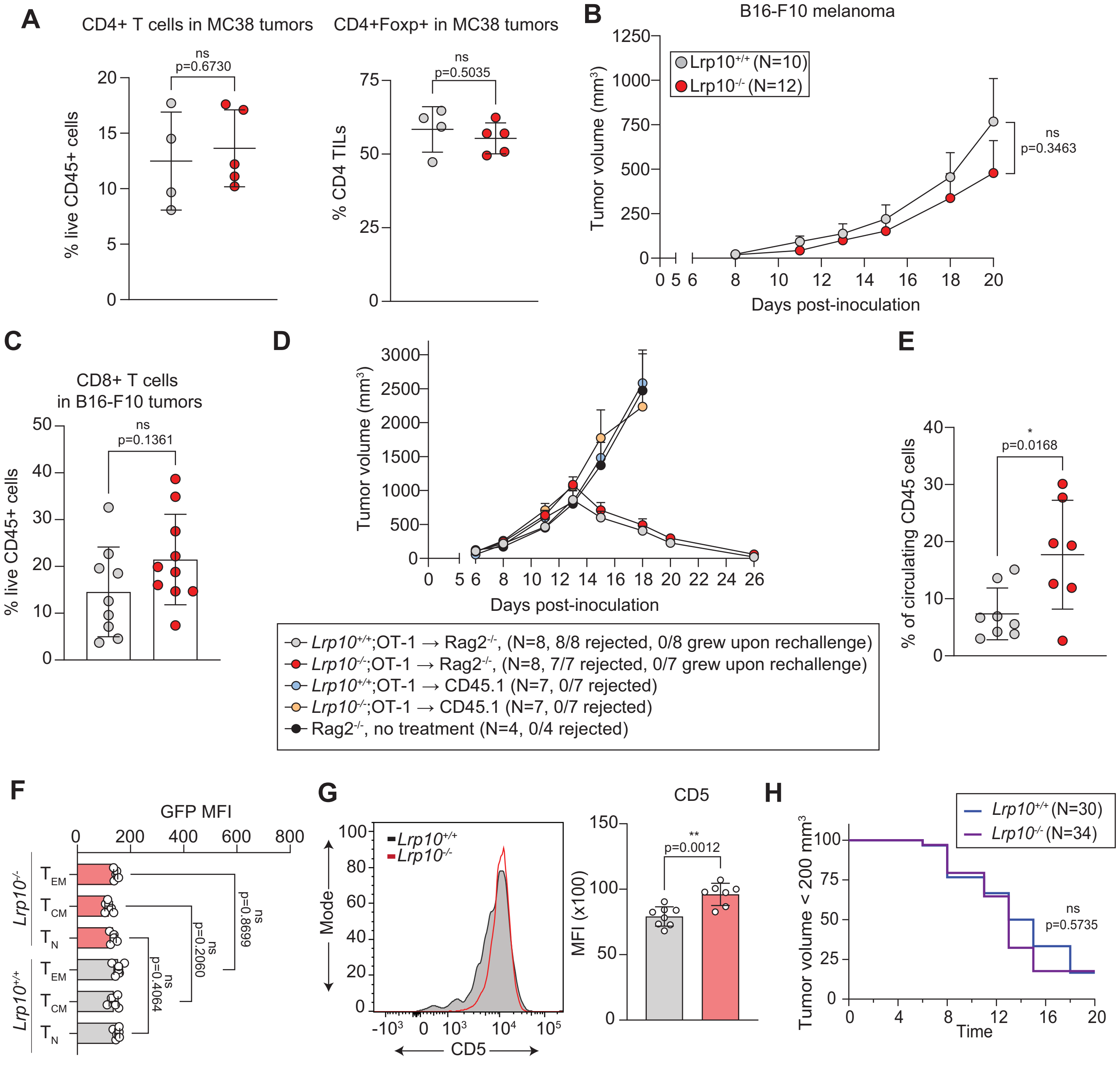

Figure EV5. Effect of *Lrp10* deletion on tumor growth in poorly immunogenic tumors and during adoptive transfer.

(**A**) Frequency of total CD4 T cells and CD4+Foxp3+ Tregs in total CD4 that were infiltrating D18 MC38 tumors on $Lrp10^{+/+}$ and $Lrp10^{-/-}$ mice. (**B**) Tumor growth of B16F10 melanoma cells injected subcutaneously into $Lrp10^{+/+}$ and $Lrp10^{-/-}$ mice. (**C**) Frequency of CD8 T cells infiltrating B16F10 tumors on D20. (**D**) Adoptive transfer of $Lrp10^{+/+}$;*OT-1* or $Lrp10^{-/-}$;*OT-1* cells into the indicated recipients on D6 after inoculation with B16-ova cells. The number of $Rag2^{-/-}$ recipients that rejected the primary tumor and were resistant to tumor rechallenge 30 days later is indicated. (**E**) The frequency of $Lrp10^{+/+}$;*OT-1* or $Lrp10^{-/-}$;*OT-1* cells in the peripheral blood of $Rag2^{-/-}$ recipients 30 days after primary tumor rejection. (**F**) GFP MFI in splenic CD8 T-cell subsets from $n = 6$ naive $Lrp10^{+/+}$;$Nur77^{GFP}$ and n = 5 $Lrp10^{-/-}$;$Nur77^{GFP}$ mice. (**G**) Representative FACS plot and CD5 MFI in T_{CM} phenotype cells from MC38 tumors on D18. (**H**) Frequency of B16F10 melanoma tumor progression in $Lrp10^{+/+}$ and $Lrp10^{-/-}$ mice treated with 10 mg/kg anti-PD1 across three separate cohorts with the indicated number n per genotype. Mice were said to have progressed if tumor volume exceeded 200 mm^3.
Data information: In bar graphs, symbols represent individual mice (biological replicates), horizontal bars indicate the mean, and error bars show SD. In (**B, D**), symbols represent the mean value and error bars show SEM. Results shown in (**A, B, D, E, G**) were replicated twice (**A, D, E, G**) or three times (**B**) in separate cohorts of at least three (**A**) or five mice (**B, D, E, G**) per genotype. Results shown in (**C**) are combined from two separate experiments. *P* values calculated with two-tailed unpaired *t* tests in (**A, B, C, E, G**), one-way ANOVA with Tukey's test (**F**), and log-rank (Mantel–Cox) test (**H**). Significant *P* values were flagged as follows: $^{*}P < 0.05$, $^{**}P < 0.01$, $^{***}P < 0.001$. *P* values > 0.05 were considered to be not significant (ns).

 © The Author(s)
